# The effect of being married on heart rate variability, an indicator of autonomic dysfunction: A retrospective study

**DOI:** 10.1097/MD.0000000000034282

**Published:** 2023-07-07

**Authors:** Alkame Akgümüş, Ahmet Balun, Tezcan Peker, Bedrettin Boyraz

**Affiliations:** a Bandirma Onyedi Eylül University, Faculty of Medicine, Cardiology Department, Balikesir, Turkey; b Mudanya University, Medicalpark Hospital, Cardiology Department, Bursa, Turkey.

**Keywords:** autonomic dysfunction, heart rate variability, marriage status

## Abstract

Heart rate variability (HRV) is a noninvasive assessment that reflects the autonomic functions of the heart and is known to be impaired in many diseases. In our study, we aimed to investigate the relationship between HRV and being married. The study included 104 patients, between the aged 20 to 40 years were included in the study. The patients were divided into 2 groups as 53 healthy married (group 1) and 51 healthy unmarried (group 2). 24-hour rhythm Holter recordings were performed on all married and unmarried patients. Group 1 had a mean age of 32 ± 5 years and 47.2% men, and group 2 had a mean age of 30 ± 5 years and 54.9% men. Standard deviation of normal to normal (SDNN) was 150 ± 40 versus 128 ± 30 (*P* = .003), SDNN index was 66 ± 20 versus 56 ± 12 (*P* = .004), the square root of the mean of the squares of the differences of the adjacent root mean square of successive differences (RMSSD) was 37 ± 10 versus 30 ± 10 (*P* < .001), percentage of successive R-R intervals that differ by more than 50 milliseconds (PNN50) was 13.5 ± 7 versus 8.5 ± 7 (*P* = .001), HF was 450 ± 270 versus 225 ± 130 (*P* < .001) found to be significantly less in the group 2. LF/HF ratio was 1.68 ± 0.65 versus 3.31 ± 1.56 (*P* < .001) found to be significantly higher in the group 2. In our study, it is possible to say that the sympathetic system effect on the heart was more dominant and the HRV was lower in the unmarried group.

## 1. Introduction

In healthy individuals, changes in the interval between heart beats in normal sinus rhythm is a normal physiological phenomenon. The main factors that determine the fluctuations between heart beats durations are respiration, thermoregulation and some baroreflex mechanisms.^[1]^ Various methods are used to evaluate autonomic functions and its effects to the cardiovascular system.^[2,3]^ The autonomic nervous system plays the main role in the regulation of the cardiovascular system.^[4]^ The autonomic functions of the heart can be evaluated by examining heart rate variability (HRV).^[3]^ HRV is defined as the cyclic variation of the time between heart beats in normal sinus rhythm. It gives information about the sympathetic and parasympathetic balance effects to the cardiovascular system. HRV is obtained by calculating the variability in R wave to R wave times on electrocardiography (ECG) to evaluate the functions of the autonomic nervous system on the heart. The dominance of the sympathetic system in relation to stress causes an increase in the heart rate and the dominance of the parasympathetic system at rest causes a decrease in the heart rate, and this balance, which works in harmony with each other in healthy people. This balance may be disrupted during the process of some diseases. Disruption of autonomic balance may lead to decreased HRV and adverse cardiovascular events.^[5,6]^ Although the heart has sympathetic and parasympathetic innervation, it is mostly under the influence of the parasympathetic nervous system, which has a negative effect on heart rate.^[7]^ Parasympathetic innervation is associated with increased HRV, while sympathetic innervation is associated with decreased HRV.^[8]^ High variability in heart rate is an important indicator of the functioning of autonomic control in healthy individuals. Conversely, epidemiological studies have shown that reduced HRV is a significant risk factor for all mortal and morbid diseases.^[9]^ Decreased HRV has been found to be associated with myocardial infarction, coronary artery diseases, heart failure, chronic mitral regurgitation, and congenital heart diseases.^[4]^

It has been shown in studies that social isolation increases gradually after adolescence, regardless of gender, especially in single male gender.^[10]^ It has been shown that chronic social isolation causes morbidity and mortality similar to risk factors such as obesity, tobacco use and hypertension.^[11]^ The overall mortality rate from loneliness and social isolation is greater than the risks from obesity and high blood presure.^[12]^ In a recent meta-analysis study, it was shown that social isolation, loneliness, and living alone increase the probability of death by approximately 30%.^[13]^ Loneliness and social isolation activate the hypothalamic-pituitary-adrenal axis and the sympathetic nervous system. Sympathetic nerve activation causes the release of lesser amounts of norepinephrine and dopamine, mainly epinephrine, into the circulation. In addition, loneliness and social isolation contribute to the risk of cardiovascular disease by leading to behavioral changes such as physical inactivity, smoking and sleep disturbance.^[14]^ In contrast, one of the most important functions of the parasympathetic nervous system is to prevent the harmful effects of over-activation of the sympathetic nervous system.^[7,15]^ While HRV is reduced in people heavily affected by sympathetic activity, HRV is increased in people with predominant parasympathetic activity.^[16]^

In our study, we examined the relationship between being unmarried and HRV, which is a significant risk factor for cardiovascular diseases.

## 2. Methods

### 2.1. Patients selection

The study included 104 patients, between the ages of 20 to 40, who applied to the cardiology policlinic of the Bandirma Training and Research Hospital, whose echocardiographic examinations were normal, and who did not have a chronic disease or psychopathological condition, and 24-hour ECG monitoring was performed. The patients were divided into 2 groups according to their marital status as married patients group 1 and unmarried patients group 2. The study was done retrospectively from hospital records. The group was included by obtaining an informed consent form. Ethical approval of the study was obtained from the local ethics committee and our study was carried out in accordance with the 1967 Helsinki declaration. Age, smoking status, alcohol consumption, marital status, drug use were evaluated. There was no difference in depression, anxiety or quality of life between marital status groups. Patients with a history of psychiatric illness or a history of psychiatric drug use were not included in the study. The patients were evaluated according to their educational status, whether they were university graduates or not. Job satisfaction was evaluated according to whether there was tension in the workplace or not. The exercise levels of the patients were evaluated according to whether they exercise for at least 30 minutes 3 days a week or not. The economic levels of the patients were evaluated as low and high according to their household incomes. The unmarried group was divided into 2 groups as those who live alone and those who do not live alone, and HRV parameters between the groups were evaluated.

### 2.2. Cardiac rhythm Holter evaluation

24-hour Holter ECG data were evaluated with a 5-lead Holter device (DMS 9800 + holter receiver version: 3.2) to evaluate HRV. Standard deviation of the RR intervals (SDNN), the mean of the standard deviations of the RR intervals over a 5-minute period (SDNN index), the square root of the mean of the squares of the differences of the adjacent RR intervals (RMSSD), the total NN interval of the number of neighboring NN intervals with a difference of more than 50 ms number (PNN50) values were calculated. High frequency band (HF-HRV), low frequency band (LF-HRV), very low frequency band values and total power were calculated as frequency dependent criteria.

### 2.3. Statistical methods

Statistical Analysis Statistical analyzes were performed using SPSS 23. The conformity of the variables to the normal distribution was examined using visual (histogram and probability graphs) and analytical methods (Kolmogorov–Smirnov/Shapiro–Wilk tests). In comparison between groups, Student *t* test was used for normally distributed parameters in independent samples and Mann–Whitney *U* test was used for non-normally distributed variables. The relationship between variables was examined using the Spearman test. Values with *P* < .05 were considered statistically significant.

## 3. Results

The study included 53 married (mean age 32 ± 5 years, 47.2% male) and 51 unmarried (mean age 30 ± 5 years, 54.9% male) who were evaluated in the cardiology policlinic of our hospital with 24-hour ECG holter monitoring. Demographic and clinical characteristics of the group 1 and group 2 are shown in Table 1. There was no statistically significant difference between the 2 groups in terms of age, gender, weight, height, body mass index, left ventricle ejection fraction, education level, work strain, household income and exercise levels. Pulse, maximum heart rate, minimum heart rate, systolic and diastolic blood pressure values were higher in the group 2 but no statistical difference was found.

**Table 1:** Demographic and clinical characteristics.

Variable	Group 1 (n = 53)	Group 2 (n = 51)	*P* value
Age, (yr)	32 ± 5	30 ± 5	.07
Male, n (%)	47.2%	54.9%	.28
Smoker, n (%)	49.1%	49%	.58
Graduated from a University, n (%)	34%	43.1%	.34
Physical activity ≥ 3 times/week, n (%)	43.4%	35.3%	.39
Work strain, n (%)	20.8%	23.5%	.73
Low household income, n (%)	56.6%	60.8%	.66
Weight, (kg)	67 ± 15	70 ± 12	.37
Height, (cm)	169 ± 10	170 ± 10	.57
Body mass index (kg/m²)	23 ± 2	23 ± 2	.22
Systolic blood pressure, (mm Hg)	119 ± 18	124 ± 15	.14
Diastolic blood pressure, (mm Hg)	73 ± 12	75 ± 10	.25
Heart rate, (beats/min)	78 ± 10	80 ± 8	.29
Max Heart rate, (beats/min)	132 ± 15	136 ± 10	.053
Min Heart rate, (beats/min)	50 ± 8	53 ± 8	.08
LVEF %	61 ± 8	62 ± 8	.88

LVEF = left ventricular ejection fraction.

When HRV parameters were evaluated (Table 2), LF 655 ± 350 versus 610 ± 250 (*P* = .46) and VLF 985 ± 425 versus 910 ± 310 (*P* = .31) less in the group 2, but no significant difference was found. SDNN was 150 ± 40 versus 128 ± 30 (*P* = .003), SDNN index was 66 ± 20 versus 56 ± 12 (*P* = .004) and RMSSD was 37 ± 10 versus 30 ± 10 (*P* < .001), PNN50 was 13.5 ± 7 versus 8.5 ± 7 (*P* = .001), HF was 450 ± 270 versus 225 ± 130 (*P* < .001) found to be significantly less in the group 2. LF/HF ratio was 3.31 ± 1.56 versus 1.68 ± 0.65 (*P* < .001) found to be significantly higher in the group 2. After the unmarried group was divided into 2 groups as those who live alone and those who do not live alone, no statistically significant difference was found when the HRV parameters between the groups were examined (Table 3).

**Table 2:** Heart rate variability parameters between groups.

Variable	Group 1 (n = 53)	Group 2 (n = 51)	*P* value
SDNN (ms)	150 ± 40	128 ± 30	.003
SDNNI (ms)	66 ± 20	56 ± 12	.004
RMSSD (ms)	37 ± 10	30 ± 10	<.001
PNN50 (%)	13.5 ± 7	8.5 ± 7	.001
HF (ms²)	450 ± 270	225 ± 130	<.001
LF (ms²)	655 ± 350	610 ± 250	.46
VLF (ms²)	985 ± 425	910 ± 310	.31
LF/HF	1.68 ± 0.65	3.31 ± 1.56	<.001

HF = high frequency band, LF = low frequency band, PNN50 = percentage of difference between consecutive NN intervals exceeding fifty milliseconds, RMSSD = square root of differences between consecutive normal NN intervals, SDNN index = average of standard deviations of all NN intervals for all 5-min segments in 24 h, SDNN = standard deviation of NN intervals, VLF = very low frequency band.

**Table 3:** Heart rate variability parameters in the unmarried group between those living alone and those not living alone.

Variable	Living alone (n = 37)	Not living alone (n = 14)	*P* value
SDNN (ms)	125 ± 31	138 ± 30	.18
SDNNI (ms)	56 ± 13	57 ± 12	.80
RMSSD (ms)	29 ± 9	31 ± 10	.38
PNN50 (%)	7.8 ± 6.4	10.6 ± 7.7	.18
HF (ms²)	222 ± 133	238 ± 132	.70
LF (ms²)	589 ± 224	673 ± 307	.28
VLF (ms²)	875 ± 317	1008 ± 281	.17
LF/HF	3.32 ± 1.68	3.28 ± 1.26	.92

HF = high frequency band, LF = low frequency band, PNN50 = percentage of difference between consecutive NN intervals exceeding fifty milliseconds, RMSSD = square root of differences between consecutive normal NN intervals, SDNN index = average of standard deviations of all NN intervals for all 5-min segments in 24 h, SDNN = standard deviation of NN intervals, VLF = very low frequency band.

## 4. Discussion

In our study, SDNN, SDDN index, RMSSD, PNN50, and HF values in unmarried and married control group patients with similar demographic and clinical characteristics were found to be significantly lower in the unmarried group; The LF/HF ratio was found to be significantly higher in the unmarried group compared to the married group. Based on this, it is possible to say that HRV in our study was lower in unmarried people.

Although many methods have been used to evaluate cardiac autonomic functions, the 24-hour Holter rhythm is frequently used in clinical practice.^[3,17]^ One of the time-dependent parameters, SDNN indicates the general status of the autonomic nervous system balance, SDNN < 50 ms was found to be associated with poor prognosis.^[16]^ In our study, it was found to be significantly lower in the unmarried group compared to the married group. The reason for the difference was thought to be less social support in the unmarried group and more sympathetic activity during the day in coping with stressors. These results support the study of Horsten et al.^[18]^

PNN50 and RMSSD predominantly reflect parasympathetic activity.^[19]^ The reason for the lower values in the unmarried group; It may be that the parasympathetic system dominates for less time due to less social support during the day against stressors. These results support the study of Randall et al.^[20]^

The HF-HRV component is activated by respiration and reflects cardiac parasympathetic activity.^[16]^ Lower HF-HRV has been associated with adverse health outcomes, including higher mortality, decreased cognitive function, depressive state.^[21]^ In our study results, it was found to be significantly lower in the unmarried group. The origin of this situation seems to be the emergence of sympathetic activity dominance, which may have decreased social interaction in unmarried people. Similarly, in the study of Smith et al, higher HF-HRV values were found in happy marriages.^[22]^

With the increase of sympathetic activity, the ratio of LF-HRV/HF-HRV also increases.^[16]^ In our study, it was found to be significantly higher in the unmarried group. It was thought that the underlying reason for this was the shift of the autonomous system in favor of the sympathetic system, especially since the unmarried group with low social support had to struggle more with stress sources. In the study of Ikeda et al, exposure to psychiatric stress such as hopelessness and helplessness was found to be higher in unmarried men and women.^[23]^

In another study, 5-year mortality was 50% in unmarried/no social support coronary heart disease patients, while 5-year mortality in the married/socially supported patient group was 18%.^[24]^ Another study showed that loneliness and social isolation increased the risk of coronary heart disease by 29% and the risk of stroke by 32%.^[25]^ In the study by Grippo et al, basal heart rate increased at baseline and after stress exposure and HRV decreased in socially isolated voles compared to socially matched voles.^[26,27]^

In our study, after the unmarried group was divided into 2 groups as those who live alone and those who do not live alone, no difference was found between the groups in the analysis. This may be because even if they do not live alone, social isolation is higher in the unmarried group and they do not interact with family members as much as married ones. It can be explained by the concept of perceived loneliness that social isolation and loneliness are not fully related to each other. It is thought that even if unmarried people live with their families, the perceived loneliness level is higher than married people, and this, in previous studies, affects the vagal regulation of perceived loneliness.^[28]^

## 5. Conclusion

When the results of our study are evaluated, there are differences in the parameters showing HRV in the unmarried group compared to the married people. Basically, we think that this difference may be due to loneliness and social isolation. From the results of the study, it is possible to deduce that the risk of developing cardiovascular adverse events is increased in the unmarried group.

Based on these results, it is possible to say that the effect of the sympathetic system on the heart was more dominant and the HRV was lower in the unmarried group in our study. Marriage can lead to a reduction in the progression of cardiovascular disease processes.

## Acknowledgments

All individuals who contributed to this publication have been included as authors. The authors did not receive support from any organization for the submitted work.

## Author contributions

**Conceptualization:** Alkame Akgümüş.

**Data curation:** Alkame Akgümüş, Ahmet Balun.

**Formal analysis:** Alkame Akgümüş, Tezcan Peker, Bedrettin Boyraz.

**Investigation:** Alkame Akgümüş, Bedrettin Boyraz.

**Methodology:** Alkame Akgümüş, Ahmet Balun.

**Project administration:** Alkame Akgümüş.

**Resources:** Alkame Akgümüş, Tezcan Peker.

**Software:** Alkame Akgümüş, Bedrettin Boyraz.

**Supervision:** Alkame Akgümüş, Ahmet Balun.

**Validation:** Alkame Akgümüş, Ahmet Balun.

**Visualization:** Alkame Akgümüş.

**Writing – original draft:** Alkame Akgümüş.

**Writing – review & editing:** Alkame Akgümüş, Ahmet Balun, Tezcan Peker, Bedrettin Boyraz.
